# Artificial intelligence in pediatric Wilms tumor imaging: diagnostic performance and the need for clinical oversight

**DOI:** 10.1590/2175-8239-JBN-2026-0083en

**Published:** 2026-06-12

**Authors:** Andre Ramos Pastor, Jesus Ravines Layza

**Affiliations:** 1 Universidad Peruana Cayetano Heredia, Facultad de Medicina, Escuela de Medicina, Lima, Peru.

To the Editor,

We read with great interest the systematic review and meta-analysis by Feitosa Filho et al.^1^ evaluating the diagnostic performance of artificial intelligence (AI) models in differentiating Wilms tumors from non-Wilms renal masses. The authors present a robust synthesis, reporting a pooled specificity of 82.8% and an area under the curve (AUC) of 0.831, highlighting the considerable potential of AI as a supportive tool in pediatric oncologic imaging^1^.

Despite these promising findings, the clinical integration of AI systems warrants careful consideration. Over-reliance on automated outputs has been associated with reduced engagement in clinical reasoning and individualized decision-making, particularly among trainees^2^. This concern is especially relevant in pediatric radiology, where developmental variability and unique patient needs demand nuanced clinical judgment. In this context, a “human-in-the-loop” framework—endorsed by recent multi-society safety statements—remains essential to mitigate algorithmic bias and ensure patient safety^3^.

Furthermore, although AI systems have demonstrated performance comparable to or exceeding that of human experts in selected pediatric oncologic imaging tasks under experimental conditions^4^, the moderate pooled sensitivity of 63.9% reported by Feitosa Filho et al.^1^ underscores current technical limitations. Rather than replacing clinical expertise, AI should be regarded as an adjunctive diagnostic aid. From an educational perspective, these findings support a collaborative model in which clinicians are trained to appraise AI-generated outputs critically in conjunction with clinical, radiological, and pathological information^2^.

In pediatric renal oncology, maintaining an appropriate balance between technological innovation and expert oversight is essential to maximize diagnostic benefit while preserving the principles of patient-centered care.

## Data Availability

No new data were generated or analyzed in this study.
