# Supplementary material for: Artificial intelligence in pediatric Wilms tumor imaging: diagnostic performance and the need for clinical oversight
Source: J Bras Nefrol. 2026 Jun 12;48(3):e20260083. doi: 10.1590/2175-8239-JBN-2026-0083en (PMC13263062; doi:10.1590/2175-8239-JBN-2026-0083en)
Supplement: Translation

# Translation: Inteligência artificial na imagem do tumor de Wilms em pacientes pediátricos: desempenho diagnóstico e a necessidade de supervisão clínica

**DOI:** 10.1590/2175-8239-JBN-2026-0083pt

**Authors:** Andre Ramos Pastor, Jesus Ravines Layza

**Affiliations:** 1 Universidad Peruana Cayetano Heredia, Facultad de Medicina, Escuela de Medicina, Lima, Peru.

Ao Editor,

Lemos com grande interesse a revisão sistemática e meta-análise de Feitosa Filho et al.^1^, que avalia o desempenho diagnóstico de modelos de inteligência artificial (IA) na diferenciação entre tumores de Wilms e massas renais não Wilms. Os autores apresentam uma síntese robusta, relatando uma especificidade combinada de 82,8% e uma área sob a curva (AUC) de 0,831, destacando o considerável potencial da IA como ferramenta de apoio na imagem oncológica pediátrica^1^.

Apesar desses achados promissores, a integração clínica de sistemas de IA requer consideração cuidadosa. A dependência excessiva de resultados automatizados tem sido associada à redução do engajamento no raciocínio clínico e na tomada de decisões individualizadas, particularmente entre profissionais em formação^2^. Essa preocupação é especialmente relevante na radiologia pediátrica, onde a variabilidade do desenvolvimento e as necessidades específicas dos pacientes exigem julgamento clínico refinado. Nesse contexto, um modelo de *human-in-the-loop* — endossado por declarações recentes de segurança de múltiplas sociedades — permanece essencial para mitigar vieses algorítmicos e garantir a segurança do paciente^3^.

Além disso, embora sistemas de IA tenham demonstrado desempenho comparável ou superior ao de especialistas humanos em tarefas selecionadas de imagem oncológica pediátrica, em condições experimentais^4^, a sensibilidade combinada moderada de 63,9% relatada por Feitosa Filho et al.^1^ evidencia limitações técnicas atuais. Em vez de substituir a expertise clínica, a IA deve ser considerada um recurso diagnóstico complementar. Do ponto de vista educacional, esses achados sustentam um modelo colaborativo no qual os clínicos são treinados para avaliar criticamente os resultados gerados pela IA, em conjunto com informações clínicas, radiológicas e patológicas^2^.

Na oncologia renal pediátrica, manter um equilíbrio adequado entre inovação tecnológica e supervisão especializada é fundamental para maximizar o benefício diagnóstico, preservando os princípios do cuidado centrado no paciente.

## Disponibilidade de Dados

Nenhum novo dado foi gerado ou analisado neste estudo.
